# Services for Connected, Cooperated, and Automated Mobility based on Big Data and Artificial Intelligence: The SHOW project paradigm

**DOI:** 10.12688/openreseurope.18878.1

**Published:** 2025-01-24

**Authors:** Georgios Spanos, Alexandros Siomos, Carolin Schmidt, Mathias Tygesen, Josep Maria Salanova, Filipe Rodrigues, Alexandros Papadopoulos, Evangelos Antypas, Athanasios Sersemis, Maria Gkemou, Antonios Lalas, Konstantinos Votis, Georgia Ayfantopoulou, Evangelos Bekiaris, Dimitrios Tzovaras

**Affiliations:** 1 Information Technologies Institute, Centre for Research and Technology-Hellas, Thessaloniki, Makedonia Thraki, 57001, Greece; 2 Hellenic Institute of Transport, Centre for Research and Technology-Hellas, Thessaloniki, Makedonia Thraki, 57001, Greece; 3 Department of Technology, Management and Economics, Technical University of Denmark, Lyngby, Capital Region of Denmark, Denmark

**Keywords:** Artificial Intelligence, Big Data, Machine Learning, Automated Mobility, Estimated Time of Arrival, Passenger Demand Prediction, Mobility Patterns Identification

## Abstract

Cooperative, Connected, and Automated Mobility (CCAM) constitutes a viable solution toward sustainable future mobility in order to achieve the target of decarbonization. Artificial Intelligence (AI) and Big Data (BD) have altered several industrial sectors providing novel and affordable solutions that facilitate and improve existing operations in these sectors. Hence, the combination of the CCAM paradigm with AI methodologies based on BD could ever increase the potential benefits of CCAM in the contemporary society. For this reason, three CCAM services, which are based on AI and BD, are introduced in the current research work in order to tackle three well-known issues of mobility such as i) the estimated time of arrival, ii) the passenger demand prediction and iii) the mobility patterns identification. The proposed CCAM services were tested on various pilot sites of the EU-funded SHOW project, thus demonstrating the potential of BD and AI in future mobility services.

## 1. Introduction

Cooperative, Connected, and Automated Mobility (CCAM) represents a transformative approach to transportation ( Guerreiro Augusto *et al.*, 2024) that emphasizes the integration of smart technologies to enhance road safety, efficiency, and sustainability. By leveraging vehicle-to-everything (V2X) communication ( Naudts *et al.*, 2021), CCAM enables real-time data exchange between vehicles, infrastructure, and traffic systems, promoting more efficient traffic management and reducing congestion ( Casademont *et al.*, 2024). This leads to lower emissions and fuel consumption, making it a crucial component in sustainable mobility strategies ( Polymeni *et al.*, 2024). Furthermore, automated driving technologies reduce human errors, improving safety while optimizing energy usage through eco-driving systems and smoother traffic flows, contributing to more environmentally friendly urban mobility solutions.

CCAM is significantly enhanced by the integration of Artificial Intelligence (AI) and Big Data (BD) ( O'Leary, 2013), creating a powerful synergy that revolutionizes transportation ( Chaalal *et al.*, 2023). AI-driven algorithms ( Brunette *et al.*, 2009) process vast amounts of data collected from vehicles, infrastructure, and sensors in real-time, enabling predictive analytics, dynamic traffic management ( De Soouza *et al.*, 2017), and adaptive driving behaviors. BD ( Sagiroglu & Sinanc, 2013) allows for the analysis of patterns in traffic flow ( Alam *et al.*, 2017), user behavior, and environmental conditions, supporting smarter decision-making in CCAM systems. This combination optimizes route planning ( Bast *et al.*, 2016), reduces congestion, improves safety, and lowers emissions, contributing to a more efficient and sustainable mobility ecosystem. Through AI and BD, CCAM becomes not only a reactive but also a proactive solution for the future of transportation. Hence, several research studies during the last years have combined BD and AI in the field of CCAM providing encouraging results related to the problems of Estimated Time of Arrival (ETA) and of Accident detection ( Antypas *et al.*, 2022; Antypas *et al.*, 2024; Papadopoulos *et al.*, 2024).

According to the aforementioned, it is clear that AI and BD could facilitate the CCAM functionality. For this reason, the purpose of this research work is to introduce novel CCAM services toward sustainable mobility, which are based on AI and BD. More specifically, as part of the SHOW EU funded research project ^1^, several AI-based services have been developed to support CCAM operations and planning decisions. **CCAM operations** are supported by providing important information to users in order to improve their travel experience and to operators in order to help them coordinate their services. This is achieved through AI-based services such as the i) *estimated time of arrival prediction for scheduled transportation*, and ii) *passenger demand prediction*. Similarly, **CCAM planning** decisions are improved through AI-based services such as the iii) *mobility patterns identification*, which help service planners design services that are tailored to the needs of the users by understanding the usage patterns revealed by the BD collected by the CCAM operators. For the aforementioned CCAM services applied in various pilot sites of SHOW, novel, sophisticated Machine Learning (ML) methodologies, proven to offer superior performance in the literature, were used such as Gradient Boosting algorithms ( Asselman *et al.*, 2023; Bentéjac *et al.*, 2021; Ke *et al.*, 2017; Prokhorenkova *et al.*, 2018), Graph Neural Networks ( Li *et al.*, 2024; Scarselli *et al.*, 2009; Wu *et al.*, 2021), Random Forest ( Aivatoglou *et al.*, 2021; Antoniadis *et al.*, 2021; Breiman, 2001), and Principal Components Analysis (PCA) ( Abdi & Williams, 2010; Greenacre *et al.*, 2022; Spanos *et al.*, 2020).

The rest of the work is organized as follows. Section 2 describes the interconnection between BD and AI services, while Section 3, Section 4 and Section 5 present the three respective services, namely, Estimated Time of Arrival Prediction for Scheduled Transportation, Passenger Demand Prediction, and Mobility Patterns Identification. Moreover, Section 6 analyzes the evaluation results from the aforementioned services, and finally, Section 7 summarizes the main findings of this research work.

## 2. BD and AI services

BD and AI services constitute indispensable parts to facilitate the CCAM operations and planning. For the smooth operation of the AI services, these receive the required data by interacting directly with the SHOW Data Management Platform (DMP) ( Sersemis *et al.*, 2021). The CCAM vehicles send data to the DMP using one of the 3 different methods described below.

1. **The pilot site provides real-time data**. The data is led to the database directly via an MQTT-broker. The KPIs are calculated automatically. As a next step, they are uploaded in the dedicated APIs in order for the connection with the SHOW Dashboard ^2^ to be feasible.
2. **The pilot site provides CSV files with historical data.** The files are uploaded once per day, week or, in the worst-case scenario, once per month. The data are led to the database by the technical team, and the procedure is completed in the same way as in the previous option.
3. **The pilot site provides Ready KPIs.** The respective files are also uploaded to the historical data platform. After this, they are loaded into the database and, omitting the calculation step, they are led to the APIs in order to be visualized in the Dashboard.

**Figure 1.**
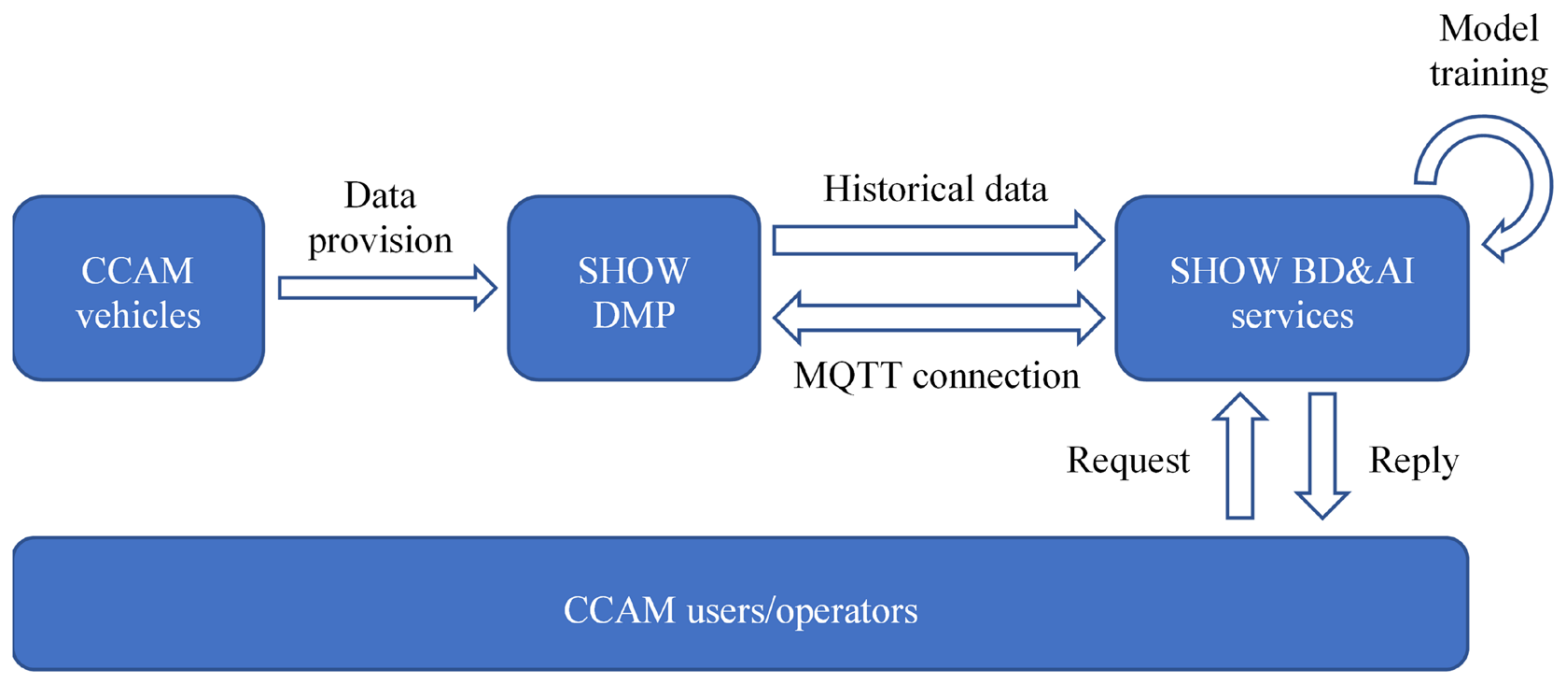
Data flow and interaction between the DMP and the BD&AI services.

The different AI services then receive historical data from the DMP, which they use to estimate the parameters of the ML models (model training). Once the ML models are trained, they are ready for deployment. At this stage, the services continue to receive real-time data from the DMP using the MQTT, in order to provide predictions to the users and CCAM operators. For services that are focused on planning rather than supporting real-time operators, such as the Mobility Patterns Identification service, the MQTT connection is unnecessary. Instead, these services focus on analyzing the available historical data and providing insights in order to, for example, improve the CCAM service or extend its operations. Figure 1 shows an overview of the data flow.

## 3. Estimated Time of Arrival for scheduled transportation

### 3.1 What it does

Estimated Time of Arrival (ETA) is a fundamental function in smart cities ( Abdi & Amrit, 2021; Altinkaya & Zontul, 2013; Singh & Kumar, 2022), as it provides information to passengers and consumers, about the status of their request. It can be utilized in both Public Transport (PT) and Demand Responsive Transport (DRT) ( Mageean & Nelson, 2003), for passengers as well as for cargo. This service provides information that can prove very useful to every kind of urban travel planning. It is, therefore, crucial, in the context of Mobility as a Service ( Butler *et al.*, 2021; Jittrapirom *et al.*, 2017; Maas, 2022) with intermodal trips for the provider to be informed of the time of arrival at the transfer points in order to better plan the route and to initiate a recalculation in the event of delays.

At the same time, accurate and reliable travel time prediction in transport networks is essential for delivering an attractive service that is able to compete with other modes of transport in urban areas. Estimation of destination arrival is of paramount importance to every transportation provider, as punctual arrival is the prevailing goal of every itinerary. The traditional application, where arrival and departure predictions are displayed on digital boards, is highly visible in the city landscape of most modern metropolises. More recently, the same information has become critical as input for smartphone trip planners in order to alert passengers about unreachable connections, alternative route choices and prolonged travel times ( Siuhi & Mwakalonge, 2016). Moreover, mobility automation and user acceptance are key values for the urban shift towards smart cities. More sophisticated Intelligent Transport Systems (ITS) ( Qureshi & Abdullah, 2013) include the predictions of connection assurance, i.e., an expert system that will decide to hold services to enable passenger exchange, in case one of the services is delayed up to a certain level. In order to operate such systems, and to ensure the confidence of passengers in the systems, the information provided must be accurate and reliable. Travel time ( Carrion & Levinson, 2012) is one of the most used performance indicators in the context of public transport systems. Travel time (or commercial speed) is generally proposed as one of the fundamental parameters for evaluating the effectiveness of the transport service. On the other hand, providing users with accurate and reliable travel forecasts can be a valid driver for attracting new demand and therefore for encouraging modal shifts towards PT services. Furthermore, a number of stakeholders are expected to benefit from the shift towards automated PT services, such as commuters, AV providers and citizens/city councils, towards Smart Cities ( Silva *et al.*, 2018) and Automation. Finally, cost savings due to automation exceed the capital cost of AVs, while assuming the shuttles are electric powered, the overall urban impact is optimistic. Considering the emergence of AVs and the surging CCAM adoption expected in the following years, the proposed ecosystem provides a cutting-edge technology of ETA calculation.

### 3.2. How it works

Raw GNSS data is pre-processed to obtain a dataset formed by segments that compose the entire bus route. Thereafter, the obtained dataset is prepared and encoded in order to be fitted to a ML model with the goal of predicting the travel time along every segment in different moments of the day. With the travel predictions for every segment that makes part of a route, the Estimated Time of Arrival between any position on the route and any given stop is computed. An expansion of the previous is to take dynamic routing into consideration. In case an itinerary has no fixed stops, or when there is low demand to a specific stop in specific time frames, the ETA to stops has to be calculated accordingly. A more sophisticated approach of ETA calculation has to consider a number of factors. Such factors include daily traffic forecasting, vehicle speed and acceleration/deceleration, real-time urban traffic, along with interaction with other objects and mixed traffic situations. Outdoor weather and specific time zoning also play an important role in ETA calculation. By utilizing the existing MQTT real-life connection within the ecosystem, real-time arrival prediction notification could be a holistic ETA service.

### 3.3. Required input data

This service only requires raw GNSS data (timestamp, latitude, and longitude of the vehicle) to work. Additionally, external data sources, such as weather information, are utilized to enhance the model. Since it uses ML models, historical GNSS data is required in order to train them. Once trained, the ETA prediction service can be deployed. To produce a prediction, the service only requires the ID of the bus and the ID of the stop for which one wants to obtain an ETA estimate. The service takes into account the current position of the bus and the travel time predictions along with the remaining route until the stop in order to output an ETA. For the expanded ETA service, weather transmission and notifications about the urban environment, along with the shuttle’s speed and acceleration are data that impact the arrival prediction.

### 3.4. Architecture

Critical to the success of the service is an advanced ML pipeline ( Singla & Malhotra, 2024) developed to continuously process observations and provide accurate arrival time predictions. We examine the process of data preprocessing and model training, the cloud architecture for deployment, and the interface that simplifies interaction for end users. Every part of the pipeline, from data input to the user interface, is designed to improve the accuracy of predictions while minimizing operational latency. This ensures that pilot sites receive timely and reliable information that enables efficient traffic management and improved passenger experience.

**Figure 2.**
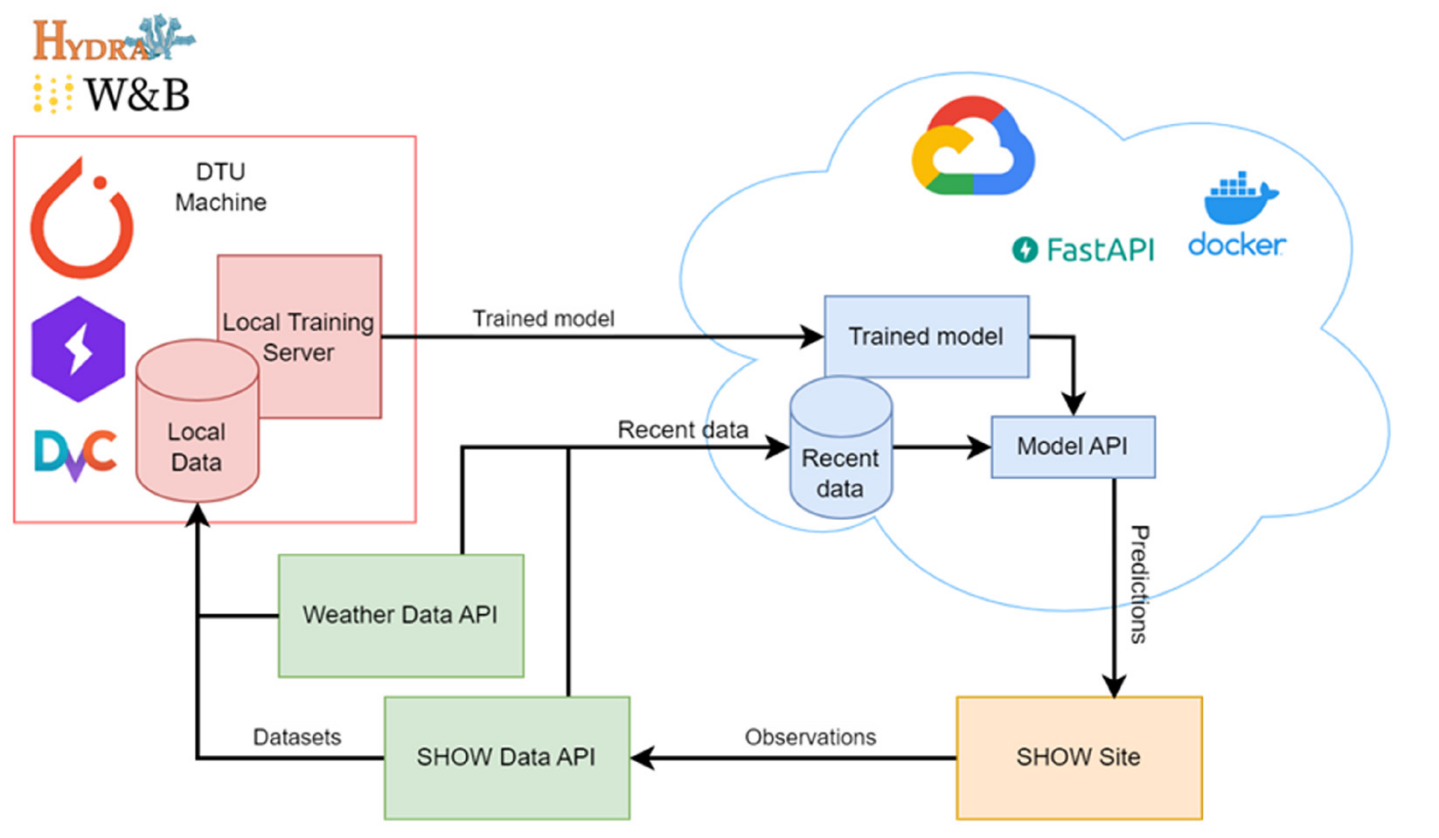
ETA architecture.

Figure 2 illustrates the training and deployment pipeline for our ML models. The workflow begins with collecting historical data via API, which is then stored as local datasets. Following data preprocessing, the models are trained on a local server. For deployment in a cloud environment, a Docker ( Acharya & Suthar, 2021) image encapsulating the model, and its associated API has been developed, allowing for seamless integration into cloud infrastructure. The architecture allows for model retraining as new data streams come in, thus ensuring continuous adaptation to evolving data patterns over time. Once trained, the models process recent data, continuously collected from API, along with up-to-date weather information. This integration enables the delivery of real-time ETA predictions, which are made available to the pilot sites on-demand via the Model API.

***3.4.1. Data preprocessing***. Ensuring a clear differentiation between run and dwell time ( Kuipers *et al.*, 2021) within public transport systems necessitates meticulous data pre-processing. We consider two scenarios: one where we have access to both speed and GNSS data, and another where we rely solely on GNSS positioning. In the first scenario, with the fusion of speed and GNSS data, we gain explicit insight into the shuttle's motion and stationary states. To ascertain dwell time, we define a shuttle as dwelling when its speed registers 0 km/h within a predetermined radius of a stop. Upon the shuttle's movement, we initiate the calculation of running time for the corresponding route segment. If the shuttle bypasses a stop without halting, we designate the dwell time as zero. However, in some scenarios where GNSS positioning might be the sole data available, determining the shuttle's movement becomes less straightforward. Here, we establish a GNSS difference threshold to identify when the shuttle halts at a stop, accounting for GNSS jitter. Dwell times are then computed based on periods of minimal movement defined by this threshold, with the stop radius remaining crucial for identification.

Specific considerations arise during pre-processing. Instances where shuttles park between runs or travel to and from depots without GNSS device shutdown require manual identification and exclusion from our dataset. Additionally, we address situations where overlapping radii occur between stops on opposite sides of the road by encoding all possible stop orders, ensuring precise calculations and eliminating ambiguity. For Graph Neural Network (GNN) utilization, data structuring involves forming a graph with nodes representing stops and edges connecting these nodes based on an adjacency matrix. Each node denotes a stop for dwell time prediction and a route segment for running time prediction, with the graph's connectivity reflecting shuttle routes. Furthermore, we enrich our dataset with hourly weather data and encode time-of-day and day-of-week features for accurate model evaluation. To prevent data leakage, a portion of the dataset is held out for testing, with variations in test selection depending on location. Since our data originates from pilot tests, careful data cleaning is essential to obtain a data set that reflects regular operation as accurately as possible.

**Figure 3.**
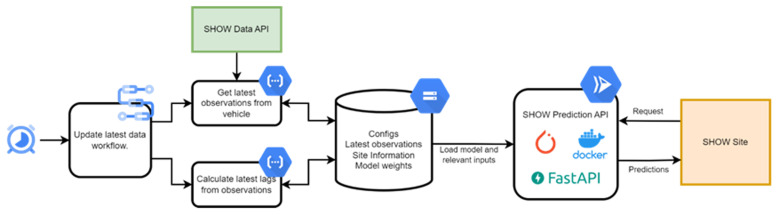
Data and API pipeline for our ETA prediction service.

***3.4.2. Interface (API)***. Utilizing the automated cloud service that regularly updates recent AVL data, the site-facing API requires only the vehicle ID and current shuttle position for processing ETA predictions. This minimalist approach greatly simplifies the user experience by focusing on the essential input, ensuring that pilot sites can quickly and easily obtain accurate ETAs without the need for complex interactions or additional data inputs. As a result, the latency from request to prediction is kept to an absolute minimum, enabling real-time, responsive service delivery to the end-users. Figure 3 shows the Data and API pipeline for our ETA prediction service.

## 4. Passenger demand prediction

### 4.1. What it does

Passenger demand prediction ( Banerjee *et al.*, 2020; di Torrepadula *et al.*, 2024) constitutes a major issue for transport operators either public or private since it plays a fundamental role in fleet management. Indeed, accurate demand forecasting in the transportation context is crucial and indispensable since it could benefit the planning of itineraries, operation scheduling, and the required resource management. Hence, this service is a valuable tool for transportation managers in order to act proactively and schedule accordingly and timely transportation issues exploiting accurate passenger demand forecasting. Considering the pilot sites in which the proposed service will be validated and applied, the service is intended initially to serve the scheduled transportation, although by performing appropriate configurations the service could also work for the case of DRT, which is also very prominent for many pilot sites.

### 4.2. How it works

Passenger demand forecasting is considered a common time series ( Esling & Agon, 2012) problem (such as stock price ( Soni *et al.*, 2022) or energy consumption ( Fumo & Biswas, 2015) prediction), since in the simplest form of this problem there is the demand variable in y axis against the time variable in x axis. It is obvious from the aforementioned that another important point that has to be taken into consideration is the level of the estimated passenger demand, namely the time that corresponds to the demand (hour, day, etc.). Bearing in mind the available data from the pilot sites, the most appropriate passenger demand forecasting for the proposed service is the daily one, which can be run every day after the integration of the most recent data. Hence, using this data, the appropriate statistical and ML algorithms could be applied in order to produce daily demand forecasting.

### 4.3. Required input data

The passenger demand forecasting service needs as data input the daily demand for each vehicle operating a different route, as well as weather data, which influences passenger demand. Therefore, in the simplest form, the date, the vehicle ID, and the cumulative passenger count are the required data. In the case of special occasions (strikes, events, national holidays, etc.) this information could be also shared in order to tune the forecast appropriately.

### 4.4. Architecture

**Figure 4.**
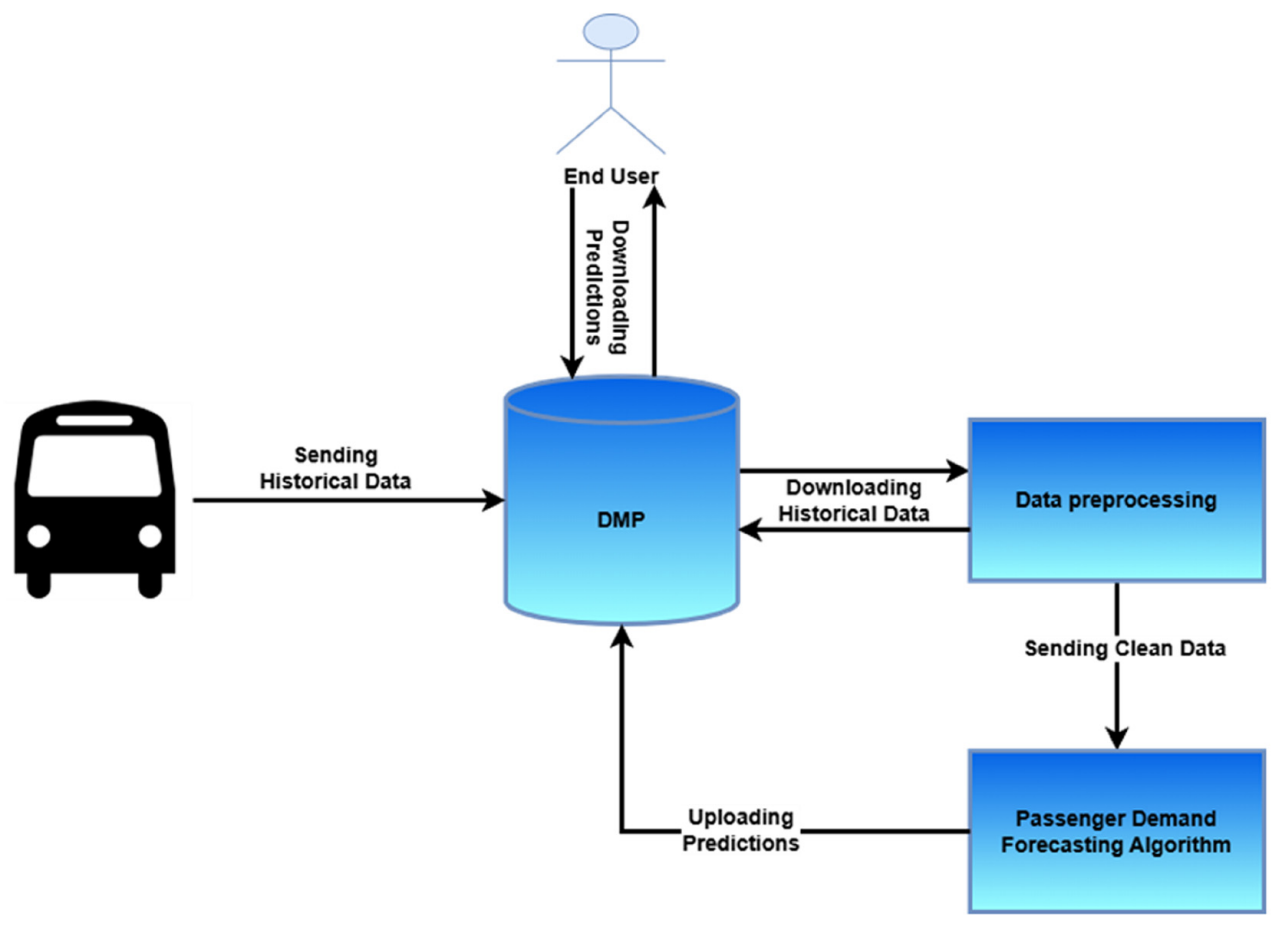
Passenger Demand Prediction Service Architecture.

The overall pipeline of the passenger demand prediction service is described in this subsection, starting from the data preprocessing of the vehicle historical data to the production of the passenger demand forecasting algorithm predictions and their uploading to the DMP. ( Figure 4).

***4.4.1.Data preprocessing***. Τhe first step of the service includes the uploading of the passenger demand historical data from the different pilot sites to the main data repository, namely the DMP. Moreover, as with all ML pipelines, an important step of the suggested service is data preprocessing. It should be noted here that as the pilot sites do not provide their historical data in the same format, a dedicated procedure was performed for each pilot site. More specifically, some pilot sites provide their dataset in the following form. For each day, a specific csv was provided, containing among others the timestamp and the cumulative passenger count in every row. Hence, in order to apply the suggested algorithm, only the date and the last value of the column number of passengers is needed. In other cases, one file contains all the required information, including a column with the specific date, accompanied by the number of passengers for that day. Finally, in other cases, a file is provided containing the trip information, namely, number of passengers, pick up and drop off place and time, thus the corresponding preprocessing was carried out.

***4.4.2. User interface***. After the data preprocessing, the next step constitutes the actual passenger demand prediction (either for one day ahead or five days ahead) using a methodology combining Random Forest and PCA. Finally, the last step includes uploading these predictions to the DMP, enabling end users to download the passenger demand predictions from a secure data platform such as the DMP. This final step was carried out for the pilot sites which evaluated the service, namely, Escrennes, Frankfurt, and Trikala.

## 5. Mobility Patterns Identification

### 5.1. What it does

Collective dynamics stemming from several individual decisions pose a great impact human mobility ( Cats, 2024). Despite the fact that individual needs and travel preferences vary greatly and the urban and regional environments in which they are located are highly diverse, there is evidence to suggest that human mobility exhibits a number of recurring characteristics over the course of time and across geographies. Disaggregate mobility data are becoming more widely available, allowing for the investigation of temporal and spatial trends ( Andrienko *et al.*, 2017) as well as the connection between microscopic behavior and subsequent aggregate flows. Advanced public transportation systems ( Hwang *et al.*, 2006) (APTS) make it possible to gather a lot of data on passenger journeys and vehicle traces. Automated vehicle location (AVL), automatic passenger counters (APC), and automatic fare collection (AFC) technologies are all included in the intelligent transportation system (ITS) component known as APTS. Through novel artificial intelligence approaches, and particularly by leveraging cutting-edge supervised or unsupervised techniques, complex spatiotemporal patterns resting on the enormous quantity of collected data may be captured. The revealed patterns can be used to provide reliable and robust short-term demand predictions and gain insights into the long-term characteristics of the passenger flows. Long-term spatiotemporal dependencies among demand data could be translated into informative visualizations based on defined thresholds and indicators enabling operators or policymakers to unfold effective strategies for meeting the demand while mitigating operating costs.

### 5.2. How it works

Based on learnt high-quality representations, mobility pattern recognition ( Ayfantopoulou *et al.*, 2022) and representation is the process of extracting spatiotemporal patterns from vast amounts of AVL, APC and AFC data. Such data keeps track of users' travels on the city's road system and includes information on their routine city mobility, including preferred routes and user group preferences. The high-quality representations are generated through representation learning, a key component of ML that automatically identifies feature patterns in the data. When given the information, the machine automatically learns the representation without the intervention of a human. Through representation learning ( Bengio *et al.*, 2013) data is projected on the latent space, where it is simpler to spot patterns and anomalies and improve comprehension of the behavior of the data as a whole. On the latent space, data samples from the spatial or temporal domain that have comparable semantic properties are projected close to one another. The data projections onto the latent space are called embeddings. Embeddings could be obtained either by supervised, semi-supervised or unsupervised techniques.

Unsupervised learning ( Hastie *et al.*, 2009) analyzes and clusters unlabeled datasets using ML techniques. Without the need for human intervention, these algorithms uncover hidden patterns or data groupings. Because of its capacity to detect similarities and contrasts in data, it is a perfect solution for exploratory data analysis ( Komorowski *et al.*, 2016), cross-selling techniques, consumer segmentation, etc. Unsupervised learning frequently seeks to uncover low-dimensional features that encapsulate some structure beneath the high-dimensional input data. Unlabeled data are grouped using clustering techniques according to their similarities or differences. Clustering algorithms are used to organize raw, unclassified data objects into groups that can be visualized as patterns or structures in the data.

Deep learning architectures ( Khamparia & Singh, 2019) based by the hierarchical architecture of the human brain system, stacking numerous tiers of learning nodes, are the primary way for feature learning in supervised and semi-supervised techniques. These architectures are often built on the distributed representation premise: observable data is generated by the interactions of many distinct components at various levels. High-quality feature representations can be extracted from intermediate layers of a specific deep neural network performing a specific task in supervised fashion, such as demand prediction. However, obtaining ground truth labels for data samples is a time-consuming and expensive process, driving to the utilization of autoencoders. Autoencoders ( Chen & Guo, 2023) are a special category of deep neural networks consisting of an encoder projecting the input data to the latent space and a decoder which reconstructs the input based on the embedding generated by the encoder. The generated embeddings capturing the semantics of data samples can be translated into meaningful static or dynamic visualizations and indicators representing the spatiotemporal mobility patterns providing insights crucial for decisions related to long-term planning of the supply of public transport services, as well as for the design of lines based on demand.

In the case of demand pattern identification APC and AFC can be harnessed. The counts of people boarding and disembarking at stations or transit lines can be obtained in one of the two ways: directly or indirectly. The former mostly employs APC devices to record passenger boardings and disembarking at a stop. APC data should contain information regarding the timestamp and location of each boarding and drop-off. While using the video monitor systems installed at the stop, image recognition technology ( Li, 2022) is another straightforward method of identifying the persons who are boarding and disembarking. Through the AFC, the number of passengers using a smart card is represented by the number of transaction records. The data does not contain passenger identity information, making it difficult to determine a specific passenger's trip route using the direct technique, which could only get passenger counts for boarding and alighting. The transaction record for the indirect approach contains the distinct ID of the smart card, which may be utilized to distinguish between the various passengers. For capturing patterns regarding routes, speeds, times of arrival and delays AVL data can be used. However, all location-based services are significantly dependent on the accurate mapping of raw GNSS trajectories onto the segments of road networks. Therefore, map matching is indispensable to accurately identify the road segments that a vehicle traveled by mitigating the prevalent discrepancies between the recorded raw GNSS trajectory and the ground truth one.

Identification of mobility patterns is solely based on historical data since it deals with the long-term spatiotemporal dynamics of the transport system. To improve the current embeddings that capture the semantics of mobility patterns, ongoing data gathering is required. This is because transport networks are highly complicated and the underlying patterns resulting from numerous individual choices are not stationary.

### 5.3. Required input data

Data generated passively and constantly, like GNSS and mobility services operations, offer significant opportunities for analyzing mobility patterns and enhancing transportation services. However, since these data are typically generated for purposes unrelated to transportation, they require processing to identify trips and mobility patterns. Existing methods for analyzing mobility patterns often rely on data from a single positioning technology, such as GNSS, which we refer to as single-sourced data. Yet, there is a lack of methods for extracting patterns and implementing multiple positioning technologies. Despite this gap, it is imperative to find methodologies that are efficient for different data sources.

Conventional mobility sensors commonly generate passively collected data, which usually consist of location and time stamps, originating as by-products of activities unrelated to transportation. In contrast, data obtained from transportation services contain valuable insights into service operations, such as vehicle occupancy or the battery status of electric scooters. Despite their distinct origins, there are analogous methodologies applicable across transportation service operations to enhance system efficiency.

Apart from data collected through individual positioning technologies like cellular or GNSS, there's an increasing volume of data stemming from the integration of multiple positioning technologies like GNSS, Wi-Fi, Bluetooth, and cellular towers. In transportation services, certain procedures exhibit similarities, such as initiating service usage through an application (e.g., purchasing a bus ticket or starting a scooter trip), allowing for the application of methods with slight adaptations.

### 5.4. Architecture

The suggested architecture for data analysis, encompassing data preprocessing, clustering analysis, pattern recognition, and visual representation, provides a robust framework for extracting valuable insights from complex datasets. By systematically applying these techniques, it is possible to uncover hidden patterns, make data-driven decisions, and ultimately drive more effective and informed actions.

***5.4.1. Data preprocessing***. Data preprocessing which is a critical step in ensuring the integrity and reliability. For the data preprocessing the following steps are adopted:

- Data Cleaning
- Feature Engineering
- Scaling & Normalization
- Encoding Categorical Variables
- Temporal Aggregation
- Spatial Aggregation
- Outlier Detection and Removal
- Dimensionality Reduction
- Data Splitting
- Data Visualization

***5.4.2. Clustering analysis***. Clustering analysis is a powerful technique for uncovering inherent structures within the data. Central to this phase is the application of the K-Means clustering algorithm ( Likas *et al.*, 2003), which is widely recognized for its efficacy in partitioning datasets into distinct groups, or clusters, based on similarity. The process begins with the critical task of optimal cluster determination, which is achieved through the Elbow method ( Bholowalia & Kumar, 2014). This method involves plotting the within-cluster sum of squares (WCSS) against the number of clusters and identifying the point at which the rate of decrease sharply diminishes—the "elbow"—indicating the optimal number of clusters. This step is essential for ensuring that the clusters are both meaningful and interpretable.

***5.4.3. Pattern recognition***. The next phase involves a comprehensive approach to pattern recognition, where both temporal and spatial dimensions of the data are analysed to extract meaningful insights. This begins with the identification of mobility patterns, where the data is scrutinized to uncover trends and correlations over time. Temporal analysis might involve examining data on an hourly, daily, or monthly basis to identify recurring patterns, such as peak travel times. In parallel, spatial analysis focuses on the distribution of activities across different geographical areas, such as identifying popular routes or commonly used stops in a transportation network.

***5.4.4. Visual data representation***. The final phase of the methodology is devoted to visual data representation, a critical component in effectively communicating the findings of the analysis. Visualizations play a pivotal role in making complex data more accessible and understandable, enabling stakeholders to quickly grasp key insights and make informed decisions. The process begins with graphical analysis, where various visual tools are employed to depict the analysed data. For instance, heatmaps ( Sobral *et al.*, 2019) can be used to show the intensity of activity across different locations, line graphs to illustrate trends over time, bar charts to compare categorical data, and scatter plots to reveal relationships between variables.

Beyond simple visualization, this phase also involves comparative analysis, where different time periods, clusters, or spatial distributions are compared to identify significant differences or trends over time. For example, comparing data from different months might reveal seasonal variations, while comparing clusters could highlight differences in user behaviour across various segments. This comparative approach provides a deeper understanding of the data and helps to identify patterns or anomalies that may warrant further investigation.

## 6. Services evaluation

This section includes the qualitative evaluation of the proposed services: Estimated Time of Arrival for Scheduled Transportation, Passenger Demand Prediction and Mobility Patterns Identification, analyzing the responses of the pilot site representatives in dedicated questionnaires for the corresponding services used in these sites. More specifically, i) ETA service was applied and evaluated in the Linköping and Tampere pilot sites, ii) Passenger Demand Prediction Service was applied and evaluated in the Frankfurt, Trikala and Escrennes pilot sites and finally, iii) Mobility Patterns Identification Service was demonstrated and evaluated in the Madrid and Frankfurt pilot sites.

### 6.1. Estimated time of arrival for scheduled transportation

In order to perform a qualitative assessment of the developed ETA prediction service, a survey was conducted with representatives from the **Tampere** and **Linköping** pilot sites. Concretely, the goals of this survey were to: i) understand the current situation at the pilot site with respect to the usage of ETA prediction, ii) understand what criteria are/should be taken into account when evaluating an ETA prediction service, iii) understand the prospects regarding ETA prediction services and potential needs from the pilot site, and iv) qualitatively evaluate the performance of the ETA prediction service developed in the scope of the SHOW project and its perceived potential for CCAM operations.

Analysing the answers from the pilot sites, both of them perceive the future of the use of AI to enhance as bright, despite one of the pilot sites currently already having their own ETA prediction service (Linköping) and the other (Tampere) not having access to such a service yet. Linköping uses a statistical modelling approach, while the proposed service uses ML techniques to predict the estimated time of arrival. Furthermore, the suggested service leverages weather data to further improve its predictions. Both pilot sites seem to agree on the use of comparisons between the actual arrival times with the predicted ones and statistical measures like Root Mean Square Error as their choice of methods to assess the accuracy of the ETA predictions, which matches exactly with the methods used to evaluate the developed service.

As a final exercise, the ETA prediction service was demoed to the pilot site responsible by showing them ETA predictions made by the service for their pilot site in different situations in comparison with the actual (observed) arrival times. The pilot site representatives were then asked to provide their subjective qualitative assessment of the predictions produced by the service, with both pilot sites classifying the predictions as “Good”.

### 6.2. Passenger demand prediction

In order to evaluate the passenger demand prediction service qualitatively a corresponding questionnaire was circulated to the three pilot sites where the service was demonstrated in real-life situations. These three pilot sites were, **Frankfurt**, **Trikala**, and **Escrennes**. The questionnaire contains questions both more generic such as the familiarization of the pilot site representatives with the problem of demand forecasting and more specific such as the performance evaluation.

Starting the analysis with the familiarization of the pilot sites with the problem, different levels of understanding are met in responses, from moderate understanding in Escrennes to excellent in Trikala. Although the aforementioned difference in problem understanding, all the respondents observe specific demand patterns in their historical data, such as weather, seasonality etc. Moreover, only in Trikala, they use current methodologies to predict demand for autonomous buses and they face specific challenges with respect to data accuracy of the historical data used in their ML methodologies.

Regarding the assessment of demand forecasting and how they can improve prediction accuracy, there is no common approach between the three pilot sites, since in Frankfurt and Escrennes there is not any strategy for assessment and improvement, while in Trikala they used evaluation metrics and reinforcement learning. However, all pilot site representatives are willing to enhance the service quality by getting feedback from drivers and passengers through questionnaires or mobile applications.

All pilot sites believe that their role is essential to improving the service by performing business analysis and software evaluation, while a training session from service providers to service operators would be useful. Finally, concerning future prospects and emerging technologies for advancing demand prediction capabilities, the use of BD, AI, external data, and simulation is considered by the respondents as of utmost importance.

### 6.3. Mobility patterns identification

The mobility patterns identification service has been applied to Madrid and Frankfurt. In order to evaluate the service offered, representatives from the two pilot sites have received the results from the aforementioned analysis with respect to the mobility patterns recognition and then answered the corresponding questionnaire.

Respondents from **Madrid** indicated a moderate understanding of this concept and how it applies to enhancing service efficiency. To identify mobility patterns within the data collected from automated vehicles, they utilize a combination of manual and automated methods. Insights gained from these analyses are primarily employed to allocate resources efficiently, ensuring that services meet user demands effectively. Data analysis for pattern recognition is conducted daily, allowing for timely adjustments and optimizations. The types of mobility patterns of most interest include fluctuations in passenger demand and traffic congestion trends, both of which are vital for operational planning and resource allocation. Despite the benefits of pattern recognition, several challenges hinder its effective implementation. Respondents noted data quality issues, and the complexities involved in algorithm implementation as significant obstacles. To overcome these challenges, collaboration with other operators and stakeholders is essential. Regular meetings and workshops, along with the use of online forums or platforms, facilitate the exchange of insights and best practices related to pattern recognition. When assessing the availability of data for pattern recognition purposes, many found it to be sufficient. However, specific data quality issues, such as inaccurate data entries, remain a concern that needs to be addressed to enhance the reliability of analyses. Feedback from pattern recognition initiatives plays a critical role in the continuous improvement of automated vehicle operations, particularly in enhancing the passenger experience. Fortunately, mechanisms are already in place to incorporate operator feedback into the refinement of pattern recognition algorithms and strategies. To further develop their proficiency in pattern recognition, many respondents expressed a desire for data analysis workshops, indicating a strong interest in continuous professional development. Looking ahead, the general sentiment regarding the future prospects of pattern recognition in automated vehicle operations is bright. Overall, respondents perceive the impact of pattern recognition on the efficiency and performance of automated vehicle operations as positive, highlighting its potential to transform the transportation landscape.

Similarly, the respondents from **Frankfurt** reported a moderate understanding of pattern recognition within automated bus operations, utilizing a combination of both manual and automated methods to identify mobility patterns. The insights gained from these techniques are primarily used to adjust bus schedules and modify routes for optimized operations. Data analysis for pattern recognition is conducted on a monthly basis, with the most sought-after mobility patterns being fluctuations in passenger demand and route efficiency. Primary challenges in leveraging pattern recognition include issues related to data quality, such as outdated information. Collaboration with other operators or stakeholders occurs regularly through meetings or workshops, although the availability of data for these purposes is considered sufficient. Despite these collaborative efforts, outdated data remains a significant obstacle, affecting the accuracy and timeliness of pattern recognition efforts. Feedback from pattern recognition initiatives is seen as vital to enhancing the passenger experience. However, there are no mechanisms currently in place to incorporate operator feedback into the refinement of pattern recognition algorithms or strategies. Many respondents expressed a need for additional training, particularly in data analysis workshops, to improve their proficiency in leveraging pattern recognition. The future prospects for pattern recognition in automated bus operations remain uncertain, though the overall impact is viewed positively, contributing to the efficiency and performance of the service.

## 7. Conclusions

As part of the SHOW project, three advanced CCAM services: i) the Estimated Time of Arrival for Scheduled Transportation service, ii) the Passenger Demand Prediction service, and iii) the Mobility Patterns Identification service, based on BD and AI have been developed, applied and evaluated in real-life situations. For the aforementioned CCAM services applied in various pilot sites of SHOW, novel and sophisticated ML methodologies were used such as Gradient Boosting algorithms, Graph Neural Networks, Random Forest, and Principal Components Analysis. All different methodologies used for the services applied in a very demanding research field, such as the automated vehicles research field, due to its immaturity and distinctiveness. However, the qualitative assessment and the feedback from different stakeholders of the pilot sites, where the services were applied and demonstrated, highlighted the significance of these services for the CCAM ecosystem as a complementary tool that could facilitate the fleet operations and planning, provide useful mobility insights, and eventually, enhance the service quality to the commuters.

## Ethics and consent

The questionnaire respondents were members of the SHOW project and their evaluations were required as part of their project role. The data collected and treated in line with the ethical guidance and agreement defined by the Ethical board of SHOW, led by the Ethical manager Dr Anna Anund and the Technical manger Dr Maria Gkemou.

## Data Availability

Siomos, A., Spanos, G., Rodrigues, F., & Salanova, J. (2024). Responses to the CCAM questionnaires [Data set]. Zenodo. https://doi.org/10.5281/zenodo.14046177 Creative Commons Attribution 4.0 International
