# Supplementary material for: Services for Connected, Cooperated, and Automated Mobility based on Big Data and Artificial Intelligence: The SHOW project paradigm
Source: Open Res Eur. 2025 Nov 27;5:24. Originally published 2025 Jan 24. [Version 2] doi: 10.12688/openreseurope.18878.2 (PMC12744199; doi:10.12688/openreseurope.18878.2)
Supplement: Peer review history

# Reviewer report: Reviewer response for version 2

**DOI:** 10.21956/openreseurope.23761.r64923

**Published:** 2025-12-27

**Authors:** Abhishek Thakur

**Affiliations:** 1 BIT Mesra, Ranchi, India

The authors have satisfactorily addressed all the comments and concerns raised during the review process. The revised manuscript demonstrates significant improvement in terms of clarity, completeness, and scholarly quality. The additions related to discussion, scalability, security considerations, and broader implications appropriately strengthen the manuscript.

 I am satisfied with the revisions made, and I believe that the manuscript now meets the scientific and editorial standards of the journal. I am happy to recommend the article for acceptance.

Is the study design appropriate and does the work have academic merit?

Yes

Is the work clearly and accurately presented and does it cite the current literature?

Partly

If applicable, is the statistical analysis and its interpretation appropriate?

Partly

Are all the source data underlying the results available to ensure full reproducibility?

Partly

Are the conclusions drawn adequately supported by the results?

Partly

Are sufficient details of methods and analysis provided to allow replication by others?

Partly

Reviewer Expertise:

Autonomous Vehicle Control, Sliding Mode Control, Model Predective Control

I confirm that I have read this submission and believe that I have an appropriate level of expertise to confirm that it is of an acceptable scientific standard.

# Reviewer report: Reviewer response for version 2

**DOI:** 10.21956/openreseurope.23761.r64924

**Published:** 2025-12-18

**Authors:** Dr. Rajkumar Singh Rathore

**Affiliations:** 1 Cardiff Metropolitan University, Cardiff, UK

Authors have addressed the queries, happy to approve

Is the study design appropriate and does the work have academic merit?

Yes

Is the work clearly and accurately presented and does it cite the current literature?

Partly

If applicable, is the statistical analysis and its interpretation appropriate?

Partly

Are all the source data underlying the results available to ensure full reproducibility?

Partly

Are the conclusions drawn adequately supported by the results?

Yes

Are sufficient details of methods and analysis provided to allow replication by others?

Partly

Reviewer Expertise:

Wireless Communications, Cyber Physical Systems/IoT, Connected and Autonomous vehicles, Intelligent Networking of Drones, AI/ML Use cases, Cybersecurity and Privacy, EV Charging Infrastructure Management, Optimization.

I confirm that I have read this submission and believe that I have an appropriate level of expertise to confirm that it is of an acceptable scientific standard.

# Reviewer report: Reviewer response for version 1

**DOI:** 10.21956/openreseurope.20428.r51029

**Published:** 2025-02-21

**Authors:** Abhishek Thakur

**Affiliations:** 1 BIT Mesra, Ranchi, India

**A. Lack of Quantitative Performance Evaluation:**

*Relevant Sections:* **Section 6 (Services Evaluation), Figures 3, 4**

- The study provides **qualitative feedback** but lacks **quantitative validation metrics** (e.g., RMSE, MAE, F1-score) for **ETA and Demand Prediction models**.
- **Recommendation:** Include a **statistical performance comparison** of the AI models, along with **error metrics and confidence intervals**.

**B. Limited Discussion on Data Privacy and Security**

*Relevant Sections:* **Section 2 (BD & AI Services), Section 5.4 (Architecture)**

- No mention of **data encryption, anonymization, GDPR compliance**, or potential **data breaches** in real-time mobility data.
- **Recommendation:** Discuss **how user data is protected**, regulatory compliance (e.g., GDPR), and **potential ethical concerns**.

**C. Scalability & Generalization to Non-EU Cities**

*Relevant Sections:* **Section 7 (Conclusions)**

- The research is limited to **EU pilot sites**, with no discussion on how these models would adapt to **different urban infrastructures** (e.g., **developing countries** or high-traffic megacities).
- **Recommendation:** Include a **scalability assessment**, or **potential modifications** needed for deployment in **diverse urban environments**.

**D. Economic and Social Impact Analysis**

*Relevant Sections:* **Section 7 (Conclusions), Section 6.3 (Mobility Patterns Identification)**

- The study does not explore **cost-effectiveness**, **public adoption challenges**, or **economic feasibility**.
- **Recommendation:** Provide a **cost-benefit analysis** comparing **AI-driven CCAM solutions vs traditional transportation models**.

**E. Figures & Diagrams Need Enhancement**

*Relevant Sections:* **Figures 1, 3, 4 (ETA Prediction & Passenger Demand Models)**

- Some system **diagrams lack clarity** and fail to illustrate **data flow or model interactions** effectively.
- **Recommendation:** Improve **visualizations** by adding **data pipelines, comparative model performance charts, and real-world deployment workflow diagrams**.

Is the study design appropriate and does the work have academic merit?

Yes

Is the work clearly and accurately presented and does it cite the current literature?

Partly

If applicable, is the statistical analysis and its interpretation appropriate?

Partly

Are all the source data underlying the results available to ensure full reproducibility?

Partly

Are the conclusions drawn adequately supported by the results?

Partly

Are sufficient details of methods and analysis provided to allow replication by others?

Partly

Reviewer Expertise:

Autonomous Vehicle Control

I confirm that I have read this submission and believe that I have an appropriate level of expertise to confirm that it is of an acceptable scientific standard, however I have significant reservations, as outlined above.

# Response

**Published:** 2025-11-17

**Authors:** Georgios Spanos

**Affiliations:** Information Technologies Institute, Centre for Research and Technology Hellas, Thessaloniki, Greece

We thank the reviewer for the detailed feedback. Below is a summary of how each comment has been addressed:

**A. Lack of Quantitative Performance Evaluation**

- **Action Taken:** We have added references to relevant studies ( *Estimated Time of Arrival* – Carolin et al., 2024; *Passenger Demand Forecasting* – Spanos et al., 2025) presenting the methodological details and performance evaluation of the AI models. These studies provide quantitative metrics (e.g., RMSE, MAE) that validate the models’ effectiveness, complementing the qualitative assessments reported in Section 6.

**B. Limited Discussion on Data Privacy and Security**

- **Action Taken:** We expanded Section 2 to include a discussion of privacy and security mechanisms. This includes data encryption, role-based access control, anonymization strategies, GDPR compliance, and a statistics-based intrusion detection system. Ethical and regulatory considerations regarding real-time mobility data are explicitly discussed. Additionally, we have added a supporting reference ( *Sersemis et al., 2023*) to further substantiate the security and privacy measures implemented in the proposed architecture.

**C. Scalability & Generalization to Non-EU Cities**

- **Action Taken:** A new discussion subsection has been added, highlighting the modular design and adaptability of the AI services. We discuss potential deployment in various urban areas beyond the SHOW project, including cities with different traffic conditions, data availability, or digital infrastructure, and provide guidance for scaling and adaptation.

**D. Economic and Social Impact Analysis**

- **Action Taken:** We included a discussion on the economic and social implications of implementing these services. Key points cover operational cost reductions, improved resource allocation, passenger satisfaction, public adoption considerations, and the potential benefits of data-driven mobility planning compared to traditional transport systems.

**E. Figures & Diagrams**

- **Action Taken:** No new diagrams were added, as the existing figures in Sections 2–5 already illustrate the system architecture, data flow, and AI service interactions adequately. As the focus of this paper is on software implementation rather than methodological performance comparison, including comparative model performance charts is not applicable. We are, however, open to assisting with any figure improvements in accordance with the guidance of the editorial board of *Open Research Europe* to ensure clarity and compliance with publication standards.

These revisions aim to address all reviewer concerns while maintaining the clarity, coherence, and scope of the SHOW project’s findings.

# Reviewer report: Reviewer response for version 1

**DOI:** 10.21956/openreseurope.20428.r50682

**Published:** 2025-02-06

**Authors:** Dr. Rajkumar Singh Rathore

**Affiliations:** 1 Cardiff Metropolitan University, Cardiff, UK

**Review of the Study**

 The article comprehensively discusses the integration of Artificial Intelligence (AI) and Big Data (BD) within the Cooperative, Connected, and Automated Mobility (CCAM) framework, specifically through the SHOW project. Three core services addressed by AI and BD methodologies are introduced: Estimated Time of Arrival (ETA) Prediction, Passenger Demand Prediction, and Mobility Patterns Identification. These services were tested across various EU locations, highlighting the potential to enhance future mobility systems.

**Strengths**

1. **Comprehensive Scope:**
  - The research thoroughly covers the rationale, implementation, and outcomes of using AI and BD to improve CCAM services through the SHOW project.
  - The project involves real-world applications and feedback, providing practical insights.
2. **Methodological Rigor:**
  - Various machine learning models are used, including Gradient Boosting, Graph Neural Networks, and Random Forest, with thoughtful discussion on their integration and purpose.
  - There is clear integration of real-time data management and AI services, considering different data types, sources, and application contexts.
3. **Evaluation and Feedback:**
  - The research reports qualitative evaluation and insights from pilot sites, offering crucial user and stakeholder feedback.
  - It captures the diverse levels of readiness and application in multiple European cities, giving a granular view of the project's utility.

**Recommendations and Areas for Improvement**

1. **Quantitative Results:**
  - The research includes qualitative assessments, quantitative results related to model performances (e.g., accuracy, RMSE for ETA predictions) would provide deeper insights into the AI models' effectiveness.
2. **Data Privacy and Security:**
  - Expanding on data privacy and protection protocols associated with using real-time vehicle and passenger data would provide a more holistic understanding of the challenges and solutions.
3. **Broad Applicability Discussion:**
  - Discussion on the applicability of the findings and methodologies to urban areas beyond the pilot sites would be beneficial to understand scalability and broader impacts.
4. **Economic and Social Impact:**
  - A more detailed analysis of the economic and social impacts of implementing these technologies could strengthen the case for widespread adoption.
5. **Graphics and Visual Aids:**
  - Including figures and diagrams (e.g., data flow diagrams or system architecture examples) in the introduction could better communicate complex interactions and methodologies.

**Approval Status**

- **Approved with Reservations **: The research presents valuable insights into the role of AI and BD in advancing CCAM, addressing the recommendations above could enhance clarity, impact, and applicability. Overall, it's a significant contribution to the field and provides a strong foundation for continued applied AI research in transportation.

Is the study design appropriate and does the work have academic merit?

Yes

Is the work clearly and accurately presented and does it cite the current literature?

Partly

If applicable, is the statistical analysis and its interpretation appropriate?

Partly

Are all the source data underlying the results available to ensure full reproducibility?

Partly

Are the conclusions drawn adequately supported by the results?

Yes

Are sufficient details of methods and analysis provided to allow replication by others?

Partly

Reviewer Expertise:

Wireless Communications, Cyber Physical Systems/IoT, Connected and Autonomous vehicles, Intelligent Networking of Drones, AI/ML Use cases, Cybersecurity and Privacy, EV Charging Infrastructure Management, Optimization.

I confirm that I have read this submission and believe that I have an appropriate level of expertise to confirm that it is of an acceptable scientific standard, however I have significant reservations, as outlined above.

# Response

**Published:** 2025-11-17

**Authors:** Georgios Spanos

**Affiliations:** Information Technologies Institute, Centre for Research and Technology Hellas, Thessaloniki, Greece

We thank the reviewer for their valuable feedback. In response:

1. **Quantitative Results / Methodological Details:** We have added details and references to relevant studies ( *Estimated Time of Arrival* – Carolin et al., 2024; *Passenger Demand Forecasting* – Spanos et al., 2025) that present the methodological part of the suggested services, thereby addressing the request for more detailed performance-related insights.
2. **Data Privacy and Security:** Additional information along with the corresponding reference (Sersemis et al., 2023) on security and privacy mechanisms has been included, highlighting the integration of role-based access control, encrypted communication protocols, and a statistics-based intrusion detection system to safeguard both vehicle and passenger data.
3. **Broad Applicability:** A discussion section has been added addressing the scalability and transferability of the proposed services to urban areas beyond the pilot sites, emphasizing modular design and adaptability to different data availability and operational conditions.
4. **Economic and Social Impact:** The new discussion section also includes an analysis of the potential economic and social benefits of deploying these services, such as improved operational efficiency, cost reduction, enhanced passenger satisfaction, and support for data-driven urban mobility planning.
5. **Graphics and Visual Aids:** No new diagrams were added, as the existing figures in Sections 2–5 already adequately illustrate the system architecture, data flows, and service interactions.

These updates aim to address all reviewer concerns while maintaining clarity and cohesion in the presentation of the SHOW project’s findings.
